# Home bitter home? Gender, living arrangements, and the exclusion from homeownership among older Europeans

**DOI:** 10.1186/s41118-016-0014-y

**Published:** 2016-11-10

**Authors:** Daniele Vignoli, Maria Letizia Tanturri, Francesco Acciai

**Affiliations:** 1 Department of Statistics, Informatics, Applications (DiSIA), University of Florence, viale Morgagni 59, 50134 Florence, Italy; 2 Department of Statistical Sciences, University of Padova, Via C. Battisti 241, 35121 Padova, Italy; 3 Department of Sociology, The Pennsylvania State University, 211 Oswald Tower, University Park, PA 16802 USA

**Keywords:** Homeownership, Gender, Living arrangements, Elderly, Europe, SHARE

## Abstract

Homeownership is the most important asset among the elderly in Europe, but very little is known about gender and living arrangement differences in this domain. This paper aims at exploring patterns of exclusion from homeownership among middle-aged and older Europeans from a gender perspective, and with a special focus on their household composition. The analysis is based on the fourth wave of the “Survey of Health, Aging and Retirement in Europe” and includes a sub-sample of about 56,000 individuals aged 50 or over, living in 16 European countries. We estimated a set of multinomial logit models to examine the probability of being either tenant or rent-free occupiers versus homeowners. Our findings show that women are generally more likely to be excluded from homeownership than men. Nevertheless, a closer look suggests that the gender gap in homeownership is essentially generated by compositional differences between men and women, with the most relevant factor being household type. Older women are almost as twice as likely as men to live alone, which is associated—other things being equal—with a particular low likelihood to be homeowners virtually in every European country.

## Introduction

The economic well-being of the elderly is often assessed using income measures, and a typical result is that older women living alone, who are generally widows, tend to display higher poverty rates than the average population (e.g. De Santis et al. 2008; Vignoli and De Santis 2008). Would the picture be any different if we also considered the assets of the elderly? Among the various types of assets, housing is often the largest component in most Western countries. Especially for older Europeans, home appears to be the most important bequeathable wealth virtually everywhere (Lefebure et al. 2006; Angelini et al. 2013a). For the aged, a home property provides a financial buffer against contingencies such as ill health or economic difficulties, and offers a nest egg for later life (Gaymu 2003). Despite between-country differences in terms of welfare state protection, from a strict economic point of view the exclusion from homeownership means the absence of the most important asset in old age.

From a gender perspective, while an extensive literature exists on women’s income and the gender wage gap, relatively little work has been done on the gender wealth or asset gap (Deere and Doss 2006). In particular, research that explicitly addresses gender-related differences in homeownership is rather scarce (Gornik et al. 2009). An exception is the paper by Blaauboer (2010), which provides an investigation on the different determinants of homeownership by gender, but limited to the case of the Netherlands and not addressing the issue specifically in later life. Most studies focusing on homeownership ignore gender, some simply omit women from the discussion, some skirt the issue by analysing homeownership patterns only for married couples, and others consider women’s homeownership, gender, or marital status as control variables, but not as crucial points of discussion. In 2006, a special issue of Feminist Economics (n. 12/2006) was entirely dedicated to the gender gap in wealth in a plurality of contexts (Deere and Doss 2006), but none of the papers has specifically focused on homeownership among older Europeans.

In parallel, existing research also highlights that home tenure is very much linked to living arrangements among the very old, and in turn this has important implications for both the well-being of the individual and the financial demands on families, on the market, and the public sector. As older individuals approach the late phase of their life, they face choices of whether to obtain home health care to allow them to maintain an independent household, move to an assisted living facility, move in with children or other family members, or move to a nursing home (Joint Center for Housing Studies of Harvard University 2014). The possibilities of choice vary considerably between men and women, across countries, but also between owners and tenants (Gaymu 2003). Previous literature finds that most homeowners do not deplete their housing wealth until very late in life after the loss of a spouse or a move into a nursing home (Venti and Wise 2004). Research that has examined the choice of living arrangements more in depth has also found that both health and wealth are strongly associated with these choices, with homeowners generally less likely to move to nursing homes and gain an independent life (Heiss et al. 2003). Hence, we might expect that women who are more likely to live alone in late life than men can be a particular fragile profile if they are not homeowner: The exclusion from property at that point may impair them to afford the out of pocket costs for both housing and home health care. Older homeowners conversely face lower monthly housing costs and therefore may have greater ability to purchase home care services compared to tenants.

Our paper explores the patterns of exclusion from homeownership among middle-aged and older Europeans from a gender perspective, with a special focus also on the type of living arrangement. The expression “exclusion from homeownership” is used for simplicity; it can also describe situations, which are not of exclusion, such as the possible choice of old individuals to bequeath their property to their children in advance, while keeping the usufruct of their home. We distinguish between gender disparities in the exclusion from homeownership arising because of differences in household compositions as well as differences within household compositions.^1^ First, we focus on the connections between gender, living arrangement, and home tenure and propose a descriptive overview of patterns of homeownership in a cross-national comparative perspective. We then look at differences in access to homeownership by gender and living arrangements in a multivariate framework. Finally, we summarize and discuss our findings.

## Background

In family demography research, the analysis of housing conditions generally focuses on homeownership (Mulder 2006a,b; Mulder and Billari 2010). This issue is important for several reasons. First of all, homeownership provides a source of income (the so-called *imputed rent*); especially in later life, homeownership is found to offer protection against poverty, as an income buffer in case of need (Castles 1998; Conley and Gifford 2006). Second, it assures future and sustainable consumption (Christelis et al. 2005). Homeowners also have the highest degree of control over their own housing conditions (Mulder and Hooimeijer 1999; Vignoli et al. 2013), as owning a home provides, for example, protection against the risk of eviction. Furthermore, by becoming a homeowner, a person has not only better economic prospects but also an enhanced quality of life (Mulder and Wagner 1998; Kurz and Blossfeld 2004). Compared with rented dwellings, owner-occupied homes are, on average, more spacious, better located and more easily adapted to a household’s needs; thus they provide better housing conditions in the long term (e.g. Mulder and Smits 1999). Moreover, homeownership is a status symbol and has an emotional value for many people (Saunders 1990). An aspect that is particularly appreciated by old people is that a property can be transferred to descendants (Kurz and Blossfeld 2004). In addition, homeownership can be considered as an alternative form of insurance that secures a valuable asset, which can be drawn upon to raise economic well-being in old age (Dewilde and Raeymaeckers 2008). Overall, homeownership is a key indicator of quality of life of the elderly from both a monetary and psychological point of view.

A home is the most important asset among older Europeans, as the proportion of household wealth accounted for by home value is more than 70 % in each country (Angelini, Brugiavini, et al. 2013a). Recent data has confirmed previous research that ownership rates decline considerably with age in most countries; however, a large part of the decline is found to be attributed to cohort effects (Lyberaki and Tinios 2005; Chiuri and Jappelli 2006; Angelini et al. 2013a). Interestingly, ownership patterns across age are quite similar across countries: an increase in homeownership is observed up to age 50–59, then levelling up, while a slight decline is noticed after age 80 almost everywhere (but not in Poland and Greece), with the exception of Denmark, Sweden, and the Netherlands where it occurs after the age of 70 (Angelini et al. 2013a). In the Mediterranean countries, where ownership acquisition has been stable for a long time and across cohorts, older cohorts show high ownership rates as well (Castles and Ferrera 1996; Kohli et al. 2005; Angelini et al. 2013b).

Within a comparative framework, variations in national housing tenure patterns can be explained by many factors: historical influences, cultural variations (e.g. in inter-generational transfers of wealth), economic cycles, housing and financial markets, institutional arrangements, and welfare state support (Lefebure et al. 2006; Poggio 2006). Variety in home tenure structure impinges on poverty differences between countries and groups (Lyberaki and Tinios 2005; Lefebure et al. 2006). An owner is in a significantly better position than a renter with the same income (Lyberaki and Tinios 2005).

Homeownership and pensions have been often considered as alternative strategies to obtain financial security in old age, as in the life cycle the costs of ownership are typically higher in early adulthood and lower at older ages (Kemeny 1981; Castles 1998). When individuals own their homes, they can rely on smaller pensions to have the same level of well-being of a renter; at a macro level, this results in a trade-off between the degree of homeownership and generosity of retirement pensions (Castles 1998). Many authors refer to this as the paradox of the “cash poor/house rich” elderly (Castles 1998; Lefebure et al. 2006). Recent studies show that the reluctance or the difficulties of old Europeans to reduce housing equity might be a relevant factor linked to their financial hardship (Angelini et al., 2009). It has been found that low-income households who are house-rich and cash-poor are more likely to sell their home in later life, but it is also noticed that changes in demographics and in living arrangements play an over-arching role in explaining home-tenure change in the final phase of the life course (Angelini et al. 2013a; Dewilde and Stier 2014). For instance, the experience of marital breakdown in adulthood is associated with a lower likelihood of being a homeowner in later life (Dewilde and Stier 2014), and this effect is stronger for women than for men (Gram-Hanssen and Bech-Denielsen 2008). Similarly, the experience of the loss of a spouse can increase the probability to move from homeownership to rent accommodation before age 65 (Angelini et al. 2013a, b). A deterioration in the health status can also have an impact on housing opportunities and choice. Those who are in poor health can have difficulties in access both the private and public housing market (Smith 1990a, 1990b). In addition, poor health status can also influence the decision to live alone or not: disability, for instance, may induce old people to live with others (e.g. in the kinship network, with a professional caregiver, in institutions, as hospitals or nursing homes) in order to get care and assistance in daily life (Weinberger et al. 1986). Finally, other things being equal, a homeowner can have more resources to purchase home care services compared to a renter, and to continue to live independently.

Socio-demographic literature indicates that a home is more than a mere asset for the elderly, and to a certain extent, it can be considered a *consumption good*. For the elderly, home represents a safe environment, rich in memories, that plays a role of refuge (Gaymu 2003). This is a further reason that may make older people particularly reluctant to sell their home and make profit by their redundant housing capacity (e.g. when children leave the nest). As mentioned earlier, homeownership is usually associated to a better quality of the home itself and to an easier social integration of the owner within a community (Kurz and Blossfeld 2004; Lyberaki and Tinios 2005). All these aspects are particularly important for the elders, as most of their everyday life takes place at home, especially in case of restrained physical mobility.

Gender is likely to play a key role in housing decisions and in homeownership. Nonetheless, a review of housing studies suggests that gender issues are typically ignored (Deere and Doss 2006); in fact, the number of studies dealing with this subject is limited, and in general, they are not specifically focused on the old population. In virtually every European country, women seem to have less access to homeownership (Kohli et al. 2005), but it is not clear whether this is linked to certain family typologies that may be more common among women or to other individual or contextual characteristics (such as limited financial resources). For instance, Warren et al. (2000), in a comparison of differentials among various asset types in Great Britain, find that housing wealth is similar for single men and women, while among unmarried parents there is a large gender disparity. In an older article by Smith (1990a, b), three barriers that women have to face when purchasing a home are identified: first, women gain lower incomes; second, they are more likely than men to live in single earner households with children; lastly, they have less access to credit—especially if they are in non-traditional family types or in non-standard employment.

Women have traditionally acquired property through marriage or inheritance (Blaauboer 2010); especially before women became active participants in the labour market, they often gained a home by marrying a man who could afford one (Deere and Doss 2006). Later, as women progressively entered the labour market, they could contribute to purchasing a home with their income; therefore, it was more common for dual-income couples to own a home than for traditional breadwinner couples. Unfortunately, very little is known about people who do not live in married couple households. A recent study illustrates that single women are less likely to be homeowners than single men (Blaauboer 2010). Within couples, the resources of men are more relevant than those of women for the process of home acquisition (Mulder and Smits 1999; Blaauboer 2010; Angelini et al. 2013b).

In Europe, the ways to access to property differs from country to country and across birth cohorts (Angelini et al. 2013b). Mulder and Billari (2010) clustered four major homeownership regimes based on the share of owner-occupied housing and access to mortgages. The first cluster they identified is the *career homeownership regime*, where homeownership is not universalistic and mortgages are widespread and a major source of homeownership finance. Here homeownership is just a step in the housing career for those with sufficient and stable incomes. Additionally, renting a home is normally seen as an acceptable alternative to property, not only for singles and childless couples but also for families. This regime includes Denmark, Luxembourg, Germany, the Netherlands, Sweden, Switzerland, and the UK. The second cluster is the *elite homeownership regime*, where homeownership is still not universalistic, but mortgages are not much widespread and housing finance has to come from savings, family help or inheritance. Consequently, homeownership is traditionally a matter for the better off. This regime includes Austria, Belgium, France, and Portugal. The third cluster is the *easy homeownership regime*, where homeownership is very much widespread and mortgages are easily accessible. This regime includes Ireland, Iceland, and Norway. Finally, a *difficult homeownership regime*, characterized by a high share of property ownership as well as a low access to mortgages, is also identified. Here inheritance is almost the only way of obtaining housing for families. In such a context, savings over the life course and inter-generational transfers seem to play a major role in the access to property. This regime includes Italy, Spain, and Greece.

In all, despite few exceptions, there is a relatively scarce literature on gender differences in home tenure. First, it is difficult to disentangle homeownership for men and women because they often live together and share the property. Second, housing studies usually use the household as the unit of analysis—ignoring intra-household issues. Besides, data on the intra-household distribution of assets is rarely collected. When women do emerge in this literature, they are identified through family type, typically as female heads of households (Deere and Doss 2006). Nevertheless, in two-adult households, the designation of the head is often arbitrary; self-reporting is likely to reflect social norms regarding who should be considered the head (Deere and Doss 2006). Previous literature suggests that using the gender of the head as a base for analysis of wealth distribution confounds marital status and gender (Deere and Doss 2006). To avoid this problem, households headed by a couple should be treated differently than households headed by individuals (Sedo and Kossoudji 2004; Blaauboer 2010). In our work, we follow this suggestion; in order to explore whether gender is associated with the exclusion from homeownership among middle-aged and older Europeans, we distinguish among different types of living arrangement.

## Dataset and variables

We use the data from the fourth wave (2011/2012) of the Survey of Health, Aging and Retirement in Europe (hereafter SHARE), which is a multidisciplinary and cross-national database of freely accessible micro data on health, socio-economic status, and social and family networks of individuals aged 50 or over (for further detail see Börsch-Supan et al. 2005). Our analysis includes about 56,000 individuals residing in 16 European countries, namely Austria, Belgium, Czech Republic, Denmark, Estonia, France, Germany, Hungary, Italy, the Netherlands, Poland, Portugal, Slovenia, Spain, Sweden, and Switzerland. The SHARE project offers a balanced sample of countries, as there are some for each European region (Northern, Southern, Eastern, and Western Europe), and therefore it provides an excellent data source to assess home-tenure patterns by gender among older Europeans, in a cross-country perspective.

For our analysis we rely on the question that asks whether an individual lives as an owner (or a member of cooperative), as a tenant (or a subtenant), or as a rent-free (a heterogeneous category including, for instance, co-residence or that home belongs to a family member who lets them live there for free). An “owner” is a respondent who lives in a home that is of property of at least one member of the household.^2^ In other words, all household members are considered owners, if at least one of them is the legal owner. For instance, if an older mother is living in the house owned by her co-resident child, she formally has no asset. The underlying assumption here is that everyone in the household equally benefits from living in a house that is owned by (at least) one of the household members. To be sure, this is probably not an issue for Northern European countries, where inter-generational co-residence is not very frequent, but it may be an issue in Southern Europe.

In the fourth wave of SHARE, there is also a question asking what percentage of the dwelling is owned by the respondent. Nevertheless, we do not consider this variable in the analysis because we believe that the cons of its usage overcome the pros. First, property and divorce laws regulating access to homeownership are likely to be different in different contexts. Second, the definition of property may be linked to different aspects than gender differences within couples per se—e.g. fiscal reasons or the wish to be entitled to get certain public benefits. For instance, when one partner is self-employed, in many contexts it is usual custom to attribute the property to the other partner, in order to avoid the repossession of goods by the authority in case of a failure of the activity. Third, it would be completely arbitrary to choose a threshold above which we consider the respondents to be “owners”, and below which they are not considered as such. Moreover, even in case we could identify this threshold, those who own only a small percentage of their home cannot be considered “rent-free” or “tenants” either. For all these reasons, we consider owners as all members who live in a home owned by the family, regardless of the amount held by each member of the family. We feel safe in this decision because for more than 80 % of homeowners, property is fully attributed to either the respondent or her/his partner. In addition, the distribution of the percentage of the dwelling owned by the respondent does not (statistically) differ between genders, which rules out a potential bias in our analyses.

## Descriptive findings

**Fig. 1:**
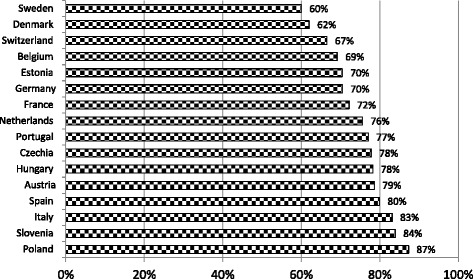
Country-specific percentage of total household wealth accounted for by home value

**Fig. 2:**
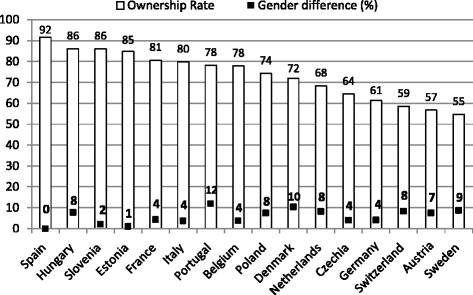
Country-specific property rate among men and gender differences

The fourth wave of SHARE data confirms that home is the most important asset among older Europeans: the proportion of household wealth accounted for by home value is more than 60 % in every country and 80 % or over in Italy, Spain, Slovenia, and Poland (Fig. 1). What it is uncertain is whether this situation is the result of pure older people preferences or rather the consequence of inadequate alternative asset and insurance market in our continent in general, and in some regions in particular, e.g. in the Mediterranean and Eastern European countries. The rate of homeownership among older Europeans varies substantially across countries; they range from 55 % in Sweden to over 80 % in the Mediterranean area (Italy, Spain, France) and in some Eastern countries (Hungary, Slovenia, Estonia) (Fig. 2). Compared to men, women are particularly disadvantaged in Portugal, Denmark, Sweden, Switzerland, the Netherlands, Hungary, and Austria (Fig. 2), where gender differences in homeownership are 8 % or more and are statistically significant at the 0.01 level. Overall, it is difficult to identify a clear regional pattern.

**Fig. 3:**
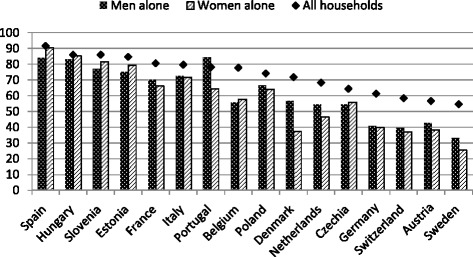
Country-specific property rate for men and women living alone, compared with the property rate for all households

As mentioned earlier, it is difficult to pinpoint which family member is the homeowner in a co-resident couple or in an enlarged family. Home can be a common possession and all the components usually benefit from it if they live in the same household. Moreover, in many cases the legal owner within the family can be chosen for different reasons. Thus, the evaluation of gender differences would be very complicated, even if the legal owner is known. A first straightforward way to compare men and women property rates in a descriptive fashion is to limit the study to one-person households. Figure 3 displays the ownership rate for households of men living alone (*dotted bars*) and women living alone (*striped bars*), while the reference line is the average calculated in each country on all households (*solid line*). Country differences in homeownership are accentuated among individuals living alone: in Germany, Switzerland, Austria, and Sweden, only 40 % of them are homeowners, whereas in other countries (Spain, Hungary, Slovenia, Estonia, and Portugal) circa 80 % of individuals living alone are homeowners. Figure 3 also shows that in most countries, gender differences among individuals living alone are negligible or even reversed; the gender gap is substantial only in a handful of countries (Portugal, Denmark, Sweden, and Switzerland) and reaches 20 percentage points only in Denmark and Portugal.

**Fig. 4:**
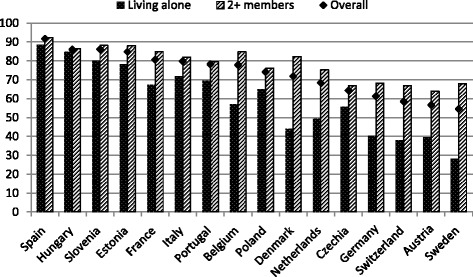
Country-specific property rate by household type

Clearly, living arrangements seem to matter more than gender in highlighting differences by property ownership. Figure 4 displays the ownership rate for households of individuals living alone (*striped bars*) and those households formed by two persons or more (*dotted bars*), while the reference line is still the average calculated in each country on all households *(solid line)*, no matter the size. Those living with other family members display higher ownership rates in all countries. The differences between individuals living alone and families formed by two members or more are particularly evident where homeownership is less widespread; differences over 20 % are recorded in Germany, Switzerland, Austria, and Sweden.

From these descriptive results, we have a first indication that women are disadvantaged in terms of home tenure. Nevertheless, differences by living arrangements seem to be more relevant than differences by gender. In the next section, our aim is to verify whether these associations still hold when controlling for other demographic and socio-economic confounders in a multivariate framework.

## Model specification

A multinomial logit model is used to contrast homeowners to both tenant and rent-free. Our key explanatory variables are gender and household composition (ego alone, couple alone, and living with relatives or others). All models use the calibrated weights provided in SHARE, which adjust for the complex survey design and for the non-response rate. The associations between home tenure, gender, and living arrangements are likely to be affected by other confounders as well. Thus, we control the estimates for several additional covariates, such as demographic, socio-economic and health status variables, and for two contextual variables, i.e. country and area of residence.

Age is divided into three categories (50–59, 60–69, and 70–85). The level of education is harmonized using the International Standard Classification of Education (ISCED), in order to make cross-national comparisons possible; and it is coded in the customary way: primary or less, secondary, and tertiary education. Educational attainment is related to parents’ socio-economic status (Marmot and Wilkinson 2006). In turn, parents’ socio-economic status is related to homeownership. Since one of the most common ways of becoming homeowners is through inheritance, by including education we indirectly control for this pathway. In addition, education can be considered as an approximation of individuals’ earning potential (Hoem et al. 2001; Kreyenfeld 2002). The other socio-economic status control that we included is income, which can derive from pensions (for retirees) or from earnings (for employed or self-employed individuals). In order to account for different household compositions, household income is transformed into equivalent income, by using the square root equivalence scale (OECD 2006); the equivalent income is then divided by the PPP (purchasing power parity) index provided in SHARE, which adjusts for different purchasing power across countries. Income is considered as the sum of different income items (respondents report separately the amount of income that derive from earnings, pensions, interests etc.) and is expressed in 2005 Euros; we use a natural logarithmic transformation in order to adjust for its skewed distribution. Our model also includes health status as operationalized by the self-rated health variable, a widely used general measure of health. This variable has five categories: poor, fair, good, very good, excellent. We also control for two contextual variables: country of residence (as a series of dummies, in a fixed-effect approach), and the area of residence, a binary variable indicating whether the respondent resides in an urban (big city, suburbs, large/small town) or rural (rural zone or village) area. The overall composition of the sample is shown in the Appendix table.

Recent simulations suggest that with large sample sizes of individuals within each country but only a small number of countries, analysts can reliably estimate individual-level effects but estimates of parameters summarizing country effects are likely to be unreliable (Bryan and Jenkins 2016). Therefore, we would need a larger number of countries to adopt a multilevel approach in our study. However, our analyses include a dummy variable indicating country of residence (in a fixed-effect approach), aimed at controlling for any country-level characteristics that might be related with homeownership. In addition, we use the Stata option “cluster”, i.e. cluster(country), which takes into account the nested structure of the data (i.e. the fact that individuals are nested within countries) and adjusts the standard errors accordingly.

Although we look at the influence of gender and living arrangements net of basic socio-economic, health, and contextual characteristics, we do not account for unobserved factors that may simultaneously affect the exclusion from homeownership and the propensity to be in a certain family status. Therefore, the scope of our analyses remains mainly descriptive.

## Model findings

Tables 1 and 2 present the results of the logistic multinomial regression models predicting the exclusion from homeownership; they include the effects of our independent variables on the likelihood of being tenant or rent-free, respectively, versus being homeowner. Results are presented in a stepwise fashion: model 1 includes only gender; model 2 adds the contextual factors; model 3 adds age, socio-economic status, and health status; model 4 adds the household type. The interpretation of results focuses on the gender differences in exclusion from homeownership by living arrangement, while we deliberately abstain from commenting on the role of the other covariates, which are intended as basic demographic, socio-economic, and contextual controls. Importantly, they all show the expected sign, providing an indirect general validation of the statistical model itself. In general, a positive coefficient indicates a higher probability of being either tenant (or rent-free) than homeowners, and vice-versa.

**Table 1:** Multinomial regression models predicting the exclusion from home ownership (tenants versus homeowners)

Variable	Categories	Model 1	Model 2	Model 3	Model 4
Gender	Man (ref.)				
	Woman	0.167***	0.192***	0.105*	−0.002
Country	Italy (ref.)				
	Austria		1.385***	1.953***	1.908***
	Germany		1.237***	1.708***	1.728***
	Sweden		0.845***	1.376***	1.335***
	Netherlands		0.868***	1.309***	1.301***
	Spain		−1.078***	−1.180***	−1.149***
	France		0.290***	0.632***	0.614***
	Denmark		0.531***	1.233***	1.208***
	Switzerland		1.685***	2.417***	2.362***
	Belgium		0.274***	0.747***	0.734***
	Czech Republic		0.347***	0.503***	0.539***
	Poland		−0.166***	−0.180***	−0.156***
	Hungary		−1.373***	−1.415***	−1.451***
	Portugal		0.081***	−0.042	0.076
	Slovenia		−0.998***	−0.769***	−0.867***
	Estonia		−1.458***	−1.455***	−1.557***
Area of residence	Urban (ref.)				
	Rural		−1.137***	−1.261***	−1.219***
Age group	50–59 (ref.)				
	60–69			−0.211**	−0.197**
	70+			−0.297***	−0.385***
Education	Primary (ref.)				
	Secondary			−0.400***	−0.379***
	Tertiary			−0.813***	−0.799***
Self-rated health	Poor (ref.)				
	Fair			−0.169***	−0.142***
	Good			−0.408***	−0.355***
	Very good			−0.706***	−0.650***
	Excellent			−0.899***	−0.832***
Income				−1.072***	−1.009***
Household composition	Couple alone (ref.)				
	Ego alone				1.005***
	With family/with others				0.313***
Constant		−1.480***	−1.672***	0.738*	0.144
Number		54,576	54,576	54,576	54,576
BIC		77,588	70,230	67,679	66,408

*p < 0.05; **p < 0.01; ***p < 0.001

**Table 2:** Multinomial regression models predicting the exclusion from home ownership (rent-free versus homeowners)

Variable	Categories	Model 1	Model 2	Model 3	Model 4
Gender	Man (ref.)				
	Woman	0.291***	0.288***	0.165*	0.090
Country	Italy (ref.)				
	Austria		0.853***	1.238***	1.295***
	Germany		−0.051	0.257**	0.356***
	Sweden		1.559***	1.922***	2.003***
	Netherlands		−1.585***	−1.239***	−1.157***
	Spain		−1.238***	−1.292***	−1.279***
	France		−0.798***	−0.542***	−0.474***
	Denmark		−0.04	0.448***	0.532***
	Switzerland		−0.404***	0.120	0.179
	Belgium		−0.555***	−0.211**	−0.143**
	Czech Republic		1.289***	1.471***	1.551***
	Poland		0.907***	0.925***	0.932***
	Hungary		0.201***	0.209***	0.205**
	Portugal		0.105***	0.014	0.095**
	Slovenia		0.135**	0.302***	0.278***
	Estonia		0.400***	0.404***	0.396***
Area of residence	Urban (ref.)				
	Rural		0.320	0.199	0.196
Age group	50–59 (ref.)				
	60–69			0.185	0.252
	70+			0.669***	0.702***
Education	Primary (ref.)				
	Secondary			−0.237	−0.226
	Tertiary			−0.440***	−0.429***
Self-rated health	Poor (ref.)				
	Fair			−0.109***	−0.085***
	Good			−0.201*	−0.182
	Very good			−0.592***	−0.573***
	Excellent			−0.559**	−0.534*
Income				−0.748***	−0.718***
Household composition	Couple alone (ref.)				
	Ego alone				0.721***
	With family/with others				0.526***
Constant		−2.581***	−2.781***	−1.594***	−2.217***
Number		54,576	54,576	54,576	54,576
BIC		77,588	70,230	67,679	66,408

*p < 0.05; **p < 0.01; ***p < 0.001

The gender gap in homeownership—i.e. the likelihood of being tenant or rent-free instead of homeowner for women with respect to men—as we might expect, is positive and significant (model 1). Nonetheless, the story is not as simple as this initial model suggests. Controlling for contextual variables and individual characteristics only marginally decreases the magnitude of the gender gap in homeownership (models 2–3), which is still significant. Model 4 shows that the gender gap in homeownership is strongly shaped and fully explained by the type of household. In particular, both for tenants and rent-free (Tables 1 and 2), the gender gap is significant only until we add the household type. Indeed, the household composition is strongly associated with the exclusion from homeownership; we found that being in a couple increases the odds of being homeowners, compared to both individuals living alone and, to a smaller extent, to extended households.

The patterns of results we present in Tables 1 and 2 (with the gender gap in homeownership disappearing after the household type has been controlled for) might not be consistent across countries. To explore this possibility, we analyse separately each country (Table 3). The bivariate association shows that women are less likely to be homeowners in all countries, with the exception of Germany, Spain, and Estonia. However, when living arrangements are controlled for, the gender gap in homeownership narrows in all countries and disappears in most of them. In addition, even in the few countries where the gender gap persists after controlling for household type, it disappears once we account for the other individual and contextual confounders.^3^ This confirms that the results from our multivariate models based on the pooled sample hold within the vast majority of the countries in our sample and show that for most countries household type is more strongly associated to homeownership than any other individual or contextual variable. However, future studies aimed at analysing the country-specific patterns of the gender gap in homeownership will be useful.

**Table 3:** Gender gap in the exclusion from home ownership (homeowners versus tenant or rent-free) by country of residence. Results of a multinomial regression model expressed in relative risk ratios (RRR)

Country	Gender gap in homeownership (RRR)					
	Tenant			Rent-free		
	Initial	Controlling for hh type	Controlling for all	Initial	Controlling for hh type	Controlling for all
Austria	1.27**	1.04	0.94	1.53***	1.41***	1.10
Germany	1.12	0.96	0.94	1.19	1.08	1.02
Sweden	1.43**	1.08	1.06	1.35*	1.15	1.09
Netherlands	1.47***	1.31**	1.10	1.38	1.10	0.87
Spain	0.81	0.77	0.76	1.12	0.98	0.90
Italy	1.40**	1.28*	1.14	1.00	0.88	0.82
France	1.23**	1.05	0.91	1.55**	1.35	1.29
Denmark	1.53***	1.27*	1.19	2.33***	1.86**	1.73*
Switzerland	1.41***	1.19*	1.08	1.22	1.09	0.84
Belgium	1.21*	1.03	0.98	1.19	1.07	1.04
Czech Republic	1.15	1.07	1.05	1.26**	1.13	1.10
Poland	1.41*	1.34	1.34	1.52**	1.44*	1.21
Hungary	1.93**	1.82**	1.47	1.67***	1.61**	1.31
Portugal	1.44**	1.25	0.97	3.29***	3.57***	2.09**
Slovenia	0.80	0.72	0.70	1.34*	1.22	1.16
Estonia	0.91	0.72*	0.80	1.07	0.90	0.89

Note: results are controlled for variables included in Tables 1 and 2

*p < 0.05; **p < 0.01; ***p < 0.001

Table 4 reports the gender-specific predicted probability of being homeowner, tenant, and rent-free. These probabilities are calculated from model 4, while setting all other variables to their reference category. From Table 4 we can see that the gender differences are quite small, or even reversed in few instances, when we compare men and women within the same household type. On the other hand, there is a remarkable difference between household types; being in a couple is associated with the highest likelihood of being homeowners, while individuals living alone are much more likely to be tenants or rent-free. Old people living in extended families are somewhat in between, as they are less likely to be homeowners than those living in a couple, but their disadvantage is not as big as that of individuals living alone. Overall, the observed gender gap in homeownership is attributable to the different likelihood of men and women of belonging to a different household type; in fact, as shown in the Appendix table, women live alone much more frequently than men, which in turn is associated with the lowest probability of being homeowner.

**Table 4:** Gender-specific predicted probability of being homeowner, tenant, and rent-free, by household type

Household type	Homeowner		Tenant		Rent-free	
	Men	Women	Men	Women	Men	Women
Couple alone	0.866	0.862	0.085	0.084	0.049	0.054
Ego alone	0.722	0.716	0.193	0.192	0.085	0.092
With family/with others	0.813	0.807	0.109	0.108	0.078	0.085

Note: results are controlled for variables included in Tables 1 and 2

## Robustness checks

Our findings would not be valid without proving their stability in a series of sensitivity checks (results not shown, but available upon request from authors). First, we ran a sensitivity analysis to see whether marital status rather than household type would produce different results. In fact, by using marital status we can look at more specific gender differences, as people living alone can be either separated or divorced, never been married, or widowed. However, we found that marital status does not modify our results; in fact, although marital status is initially associated with homeownership, its effect tends to overlap with the effect of household type. Ultimately, the use of the “marital status” variable as an alternative to the “household type” variable poses problems related to the diverging rules governing homeownership within married and unmarried cohabiting couples. Hence, we privileged the living arrangement as a covariate in the presented models.

Second, we already acknowledged that it is not clear how homeownership is attributed in couple households. A central issue regarding gender is thus what happens in single-person households. To address this issue, we ran additional analyses where we looked at the gender gap *within* each household type (couple alone; ego alone; with family/others). These results are entirely consistent with our findings; gender differences are not statistically relevant when examined within the same household type.

Lastly, our findings remained virtually unchanged using alternative measures of either health status (self-rated limitations in daily activities, instead of self-rated health status) or financial hardship (a measure of relative poverty, instead of income).

## Concluding remarks

Although it is well-established that homeownership is one of the most important assets among older Europeans, so far very little was known about gender differences in a cross-country perspective. Our analysis, exploratory in scope, is a first effort to shed light on this topic by disentangling the effect of gender and living arrangements. We explore whether women are disadvantaged in terms of homeownership compared to men, and whether this effect is shaped by compositional differences, in terms of individual characteristics, household structure, and contextual variables.

Overall, women tend to be disadvantaged in terms of home tenure in most European countries, with wider gender differences in Northern countries than in the Mediterranean, Central, and Eastern countries. Nevertheless, differences in homeownership by living arrangements are more notable than differences by gender in all countries. The above descriptive findings have been confirmed by results of our multivariate analysis with only few minor exceptions (see Table 3).

A multinomial regression model was used to look at differences in the exclusion from homeownership by gender and living arrangements, distinguishing between tenants and rent-free, while controlling for a plurality of covariates, relative to demographic background, health and socio-economic status, as well as country and area of residence. As expected, we found that women are more likely to be excluded from homeownership than men. The household type, however, strongly mediates the gender gap in homeownership; in fact, individuals living alone are much more likely to be tenant or rent-free, and at the same time women are more likely to be living alone. In short, controlling for the household type, the gender gap in homeownership vanishes virtually in every country. Importantly, when the gender gap persists after controlling for household type, it disappears once we control for the other individual and contextual factors.

In all, we found that the gender gap in homeownership among older Europeans is essentially generated by compositional differences between men and women, with the most relevant factor being household type. Since a home is the most important asset among older Europeans, we believe this analysis raises important questions about family change and homeownership in post-industrial societies. A longer life expectancy combined with a lower propensity to re-partner makes women almost as twice as likely as men to live alone at old age. Our findings showed that this condition is associated with exclusion from homeownership in late life, thus adding an additional source of economic hardship to the lives of the elderly—particularly women—in Europe. Respondents suffering from disability or in bad health are less likely to afford housing and care at home at the same time, and therefore they can be impaired in independent living, unless subsidized housing is provided by opportune public policies. Further research should go beyond our findings, utilizing better data containing intra-household longitudinal information on homeownership as well as the process of home acquisition.

## Appendix

**Table 5:** Descriptive statistics for men and women. Means for continuous variables and percentages for categorical variables

	Male (*N* = 25,605)	Female (*N* = 30,773)
Age (%)		
50–59	37.0	32.2
60–69	32.7	29.6
70+	30.3	38.2
Education (%)		
Primary	36.5	50.0
Secondary	41.8	33.4
Tertiary	21.7	16.6
Home ownership (%)		
Owner	76.5	72.1
Tenant	17.5	20.0
Rent-free	6.0	7.9
Household type (%)		
Couple alone	50.5	41.3
Ego alone	15.3	29.1
Couple with family/others	34.2	29.6
Income (€)	20,358	17,525
Self-rated health (1–5)	2.8	2.6
Area of residence		
Urban	68.7	70.3
Rural	31.3	29.7

## Footnotes

1 The terms “living arrangement” and “household composition” will be used interchangeably.

2 Unfortunately, homeownership focuses only on the currently occupied dwelling. This definition has a caveat: It does not allow us to identify as owner those who currently live in a rented apartment, but own a house elsewhere.

3 There are few exceptions for the rent-free categories, whereby in Denmark, Hungary, and Portugal, women are still at a disadvantage.
